# Percutaneous Closure of a Diagonal Artery to Pulmonary Artery Fistula

**DOI:** 10.1016/j.jaccas.2025.103521

**Published:** 2025-06-04

**Authors:** Luai Madanat, Robert D. Safian, Marina Maraskine, Adam Tawney, Justin Trivax

**Affiliations:** Department of Cardiovascular Medicine, William Beaumont University Hospital, Corewell Health East, Royal Oak, Michigan, USA

**Keywords:** computed tomography, coronary angiography, myocardial ischemia, percutaneous coronary intervention

## Abstract

Coronary artery fistulas are uncommon anomalies that are typically asymptomatic and incidentally identified through coronary angiography or computed tomographic angiography. In this clinical vignette, we report a symptomatic patient with exertional angina and anterior ischemia on exercise stress testing. Coronary angiography revealed a fistula between the proximal diagonal artery and the main pulmonary artery. Coronary computed tomographic angiography provided precise anatomical detail, facilitating procedural planning. The patient underwent successful coil embolization of the fistula, resulting in complete resolution of angina.

## Graphical Abstract

**Figure:**
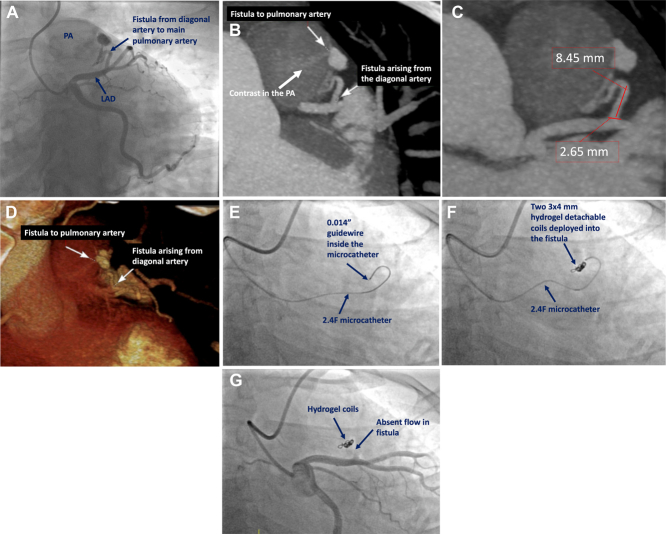

## Case Summary

**Figure 1:**
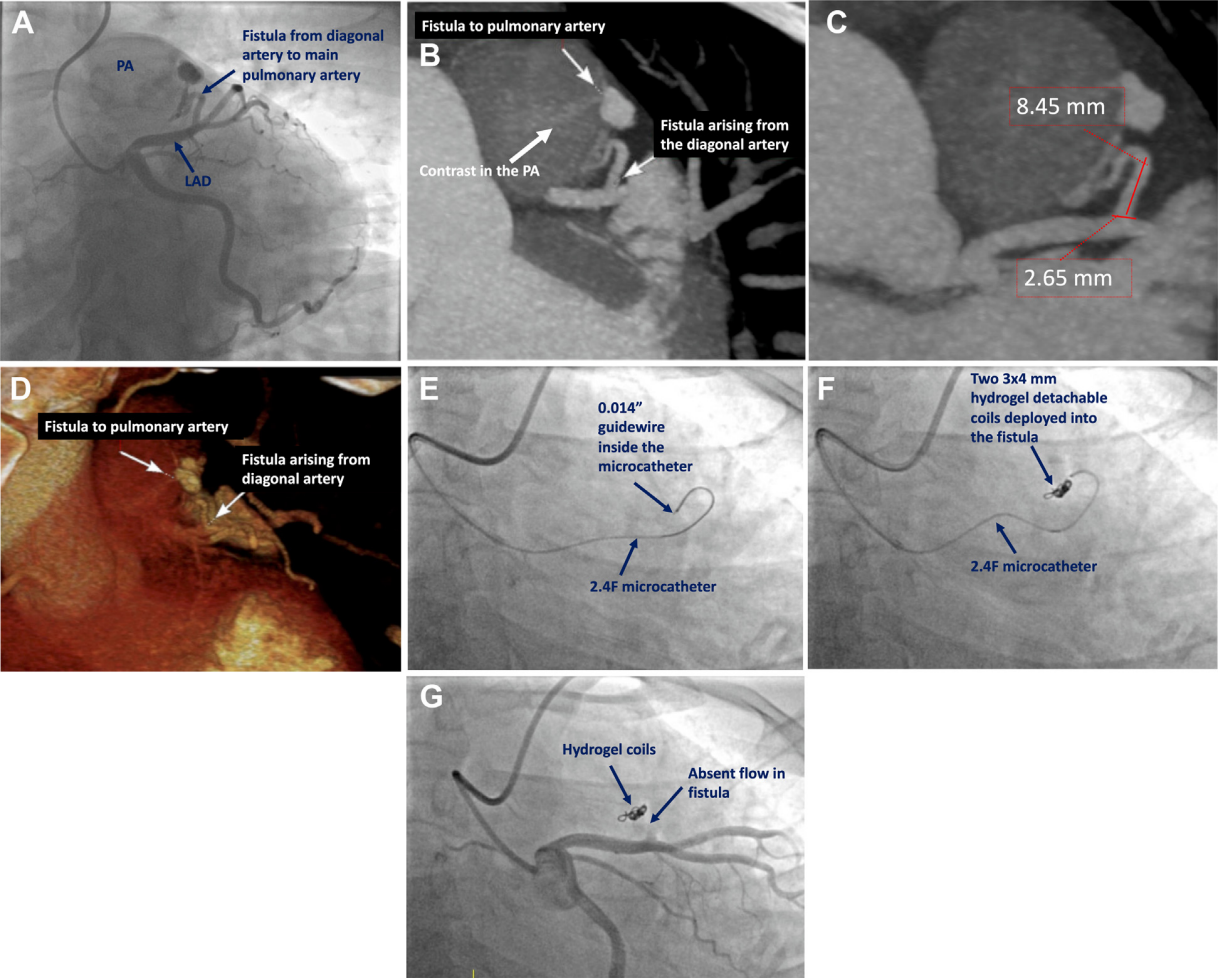
Preprocedural Imaging and Transcatheter Intervention (A) Coronary angiography demonstrates a fistula from the diagonal artery to the pulmonary artery. (B) Coronary computed tomographic angiography demonstrates the fistula from the diagonal artery to the pulmonary artery. (C) Measurements of the length (8.4 mm) and diameter (2.6 mm) of the fistula are used to guide selection of coils for embolization. (D) Three-dimensional volume rendering demonstrates the origin and course of the fistula. (E) The 2.4-F microcatheter is advanced over a 0.014-inch guidewire into the fistula. (F) Two 3 × 4 mm hydrogel detachable coils are deployed into the fistula. (G) Final angiography demonstrates occlusion of the fistula and TIMI flow grade 3 in the left anterior descending coronary artery and diagonal artery.

A 42-year-old woman was referred for coronary angiography for evaluation of exertional angina and exercise-induced anterior ischemia. Coronary angiography identified a fistula from the proximal diagonal artery to the main pulmonary artery (Figure 1A), with reduced flow in the distal left anterior descending coronary artery (Video 1). Other coronary arteries and left ventricular function were normal. Coronary computed tomographic angiography (CTA) confirmed a fistula arising from the proximal diagonal branch to the main pulmonary artery, measuring 2.6 × 8.4 mm (Figures 1B to 1D). Heart team consensus was to proceed with coil embolization of the fistula. Using right radial artery access, a 6-F guiding catheter engaged the left main coronary artery. A 2.4-F microcatheter was advanced into the fistula over a 0.014-inch guidewire (Figure 1E), and two 3 × 4 mm hydrogel detachable coils (AZUR CX, Terumo Interventional Systems) were deployed (Figure 1F). Final angiography confirmed successful closure of the fistula (Figure 1G, Video 2). The patient experienced complete resolution of angina after intervention.

**Take-Home Messages**

• Despite their small size, CAFs can cause significant symptoms by inducing myocardial ischemia through mechanisms such as blood shunting and coronary steal.
• This case underscores the critical role of CTA in accurately assessing fistula anatomy, optimizing procedural strategy, and guiding appropriate coil size for embolization.

Coronary artery fistulas (CAFs) are rare coronary anomalies, present in <0.5% of the population. Most CAFs are incidental findings during coronary angiography or CTA.^1^ Although most patients with CAFs are asymptomatic, some CAFs may cause intracardiac shunting or coronary steal, leading to ischemia. There are limited data to guide the management of CAFs, and many recommendations are drawn from small case studies or expert opinion.^2^ The decision to treat this patient with coil embolization was driven primarily by angina and objective myocardial ischemia on stress testing, which corresponded to the anatomical location of the fistula. Precise fistula dimensions were obtained using coronary CTA, and the coil diameter was selected to be 20% to 30% oversized compared with the fistula diameter to avoid coil migration.

## Funding Support and Author Disclosures

The authors have reported that they have no relationships relevant to the contents of this paper to disclose.

## Appendix

Video 1 Coronary Angiography Demonstrates a Fistula Between the Diagonal Artery and the Main Pulmonary Artery and Reduced Flow in the Distal Left Anterior Descending Coronary Artery

Video 2 Angiography After Coil Embolization Demonstrates Occlusion of the Fistula and Improved Flow in the Distal Left Anterior Descending Coronary Artery
